# Staphylococcal species heterogeneity in the nasal microbiome following antibiotic prophylaxis revealed by *tuf* gene deep sequencing

**DOI:** 10.1186/s40168-016-0210-1

**Published:** 2016-12-02

**Authors:** Claire L. McMurray, Katherine J. Hardy, Szymon T. Calus, Nicholas J. Loman, Peter M. Hawkey

**Affiliations:** 1 Heart of England NHS Foundation Trust, Birmingham Heartlands Hospital, Bordesley Green East, Birmingham, B9 5SS UK; 2 Public Health England Birmingham Laboratory, Birmingham Heartlands Hospital, Bordesley Green East, Birmingham, B9 5SS UK; 3 Institute of Microbiology and Infection, School of Immunity and Infection, The College of Medical and Dental Sciences, The University of Birmingham, Birmingham, B15 2TT UK; 4 Present address: Infrastructure and Environment Research Division, School of Engineering, University of Glasgow, Glasgow, G12 8QQ UK

**Keywords:** *tuf* gene, Staphylococcus, *S. aureus*, *S. epidermidis*, Nose, Surgical prophylaxis, Antibiotics

## Abstract

**Background:**

Staphylococci are a major constituent of the nasal microbiome and a frequent cause of hospital-acquired infection. Antibiotic surgical prophylaxis is administered prior to surgery to reduce a patient’s risk of postoperative infection. The impact of surgical prophylaxis on the nasal staphylococcal microbiome is largely unknown. Here, we report the species present in the nasal staphylococcal microbiome and the impact of surgical prophylaxis revealed by a novel culture independent technique. Daily nasal samples from 18 hospitalised patients, six of whom received no antibiotics and 12 of whom received antibiotic surgical prophylaxis (flucloxacillin and gentamicin or teicoplanin +/− gentamicin), were analysed by *tuf* gene fragment amplicon sequencing.

**Results:**

On admission to hospital, the species diversity of the nasal staphylococcal microbiome varied from patient to patient ranging from 4 to 10 species. Administration of surgical prophylaxis did not substantially alter the diversity of the staphylococcal species present in the nose; however, surgical prophylaxis did impact on the relative abundance of the staphylococcal species present. The dominant staphylococcal species present in all patients on admission was *Staphylococcus epidermidis*, and antibiotic administration resulted in an increase in species relative abundance. Following surgical prophylaxis, a reduction in the abundance of *Staphylococcus aureus* was observed in carriers, but not a complete eradication.

**Conclusions:**

Utilising the *tuf* gene fragment has enabled a detailed study of the staphylococcal microbiome in the nose and highlights that although there is no change in the heterogeneity of species present, there are changes in abundance. The sensitivity of the methodology has revealed that the abundance of *S. aureus* is reduced to a low level by surgical prophylaxis and therefore reduces the potential risk of infection following surgery but also highlights that *S. aureus* does persist.

**Electronic supplementary material:**

The online version of this article (doi:10.1186/s40168-016-0210-1) contains supplementary material, which is available to authorized users.

## Background

Staphylococci are ubiquitous commensals of the human skin and mucous membranes and are a major cause of hospital-acquired infections. Culture independent studies using the 16S rRNA gene have identified staphylococci as a major constituent of the nasal microbiome in both healthy individuals [1, 2] and hospitalised patients [3]. The staphylococcus genus consists of 52 species and 28 subspecies; however, culture independent studies using the 16S rRNA gene have only been able to identify *Staphylococcus aureus* from the staphylococcus genus, due to high sequence similarity of the other staphylococcal species. Other taxonomy genes including *hsp60, sodA*, *rpoB*, *gap* and *tuf* have been shown to be more discriminative than 16S rRNA gene at identifying staphylococcus species [4]. The *tuf* gene encodes the elongation factor Tu protein, which is involved in peptide chain formation. DNA sequencing of the *tuf* gene on a panel of reference strains has shown that it can discriminate all staphylococcal species and identification is comparable to MALDI-TOF [5].

In surgical patients, postoperative infection such as surgical site infections (SSI) and prosthetic joint infections (PJI) cause a substantial increase in morbidity and mortality. Staphylococci are the most common causative agent, with 20% of SSI cases caused by *S. aureus* [6] and 30–43% of PJI cases caused coagulase negative staphylococci (CNS) [7]. Antibiotic surgical prophylaxis is administered to surgical patients to reduce the risk of postoperative infection. The only culture-dependent study of the impact of antibiotic surgical prophylaxis on the nasal microbiota [8] reported that antibiotics did impact on staphylococcal diversity but that the effect was patient specific. However, a major limitation of the study was the culture-based methodology.

The aim of this study was to investigate the nasal staphylococcal microbiome during antibiotic prophylaxis using *tuf* gene fragment amplicon sequencing, a novel culture independent methodology.

## Methods

### Study design, population, nasal sampling and swab processing

Patients over the age of 18 years were recruited within 24 h of hospitalisation, to either one of the surgical or cardiology wards at the Heart of England NHS Foundation Trust via written consent. Patients were excluded if hospitalised for greater than 24 h or if the surgical procedure prohibited nasal sampling pre- and/or postoperatively. Ethical approval for the study was gained from the NRES Committee West Midlands (08/H1206/133). On recruitment, a nasal sample was taken using a flocked transport eswab (Sterilin, UK) within 24 h of admission, and prior to the administration of any antibiotics. Subsequently, nasal samples were taken daily, with all the patients that received prophylactic antibiotics receiving them after the first and prior to the second sample being taken.

Nasal sampling was carried out by a standardised procedure, with all samples being taken by rotating the swab three times applying a medium pressure to each nostril in turn, and then placing in the transport medium, and transported back to the laboratory to be processed. Swabs were vortexed for 10 s in the 1 ml liquid Amies transport media, 200 μl used for conventional culture and 800 μl stored at −80 °C for *tuf* gene fragment amplicon sequencing.

For all recruited patients, clinical and demographic data was collected including age, sex, antibiotic prophylaxis received, previous antibiotic usage and previous hospital admission in the last 6 months.

### Staphylococcal species identified by conventional culture

Staphylococci were cultured on selective media and identified to species level as described previously [8]. In brief, *S. aureus* and coagulase negative staphylococci (CNS) isolates were preliminary identified by colony morphology from culture media. Multiple representative isolates of *S. aureus* and CNS were selected based on the total colony count of each bacterium using the following criteria: >50 colonies five isolates, between 10 and 50 three isolates, and <10 colonies one isolate. Isolates were speciated by PCR and MALDI-TOF (Bruker Daltonik GmbH, Bremen, Germany).

### *Tuf* gene fragment amplicon sequencing

Frozen nasal samples were thawed, a 400-μl volume added to lysing matrix B beads (MP Biomedicals UK, Cambridge, UK) and homogenised at 6500 rpm for 30 s using the MagNA lyser instrument (Roche Applied Science, Penzberg, Germany). Samples were then centrifuged at 3000 rpm for 2 min, and the supernatants containing DNA material stored at −20 °C.

Amplicon sequencing targeted a 412-bp staphylococcus specific region of the *tuf* gene using previously published primers [9], with the inclusion of a heterogeneity spacer, Illumina adapter and linker [10] (see Additional file 1: Table S1). The initial PCRs of the *tuf* gene fragment were performed using the aforementioned primers in triplicate and pooled. Subsequently, samples were cleaned up, and an index PCR performed to add sample specific indices and Illumina sequence adapters using the Nextera XT index kit, as described previously [11]. DNA was quantified by Qubit 2.0 flurometer (Invitrogen, California, USA), normalised to 0.1 ng/μl and pooled to create amplicon library, which was quantified by qPCR using the Library Quantification Kit for Illumina (Kapa Biosciences, Woburn, MA, USA). For sequencing, PhiX Control library (v3) (Illumina) was added to the amplicon library at 20%. Library pool was sequenced using paired-end 300 cycles V3 Illumina Miseq reagent kit on an Illumina Miseq sequencer (Illumina, San Diego, CA, USA).

### Illumina Miseq data processing and analysis

Initial quality filtering of raw sequence reads, construction of contigs and subsampling of 5000 representative sequences per sample were carried out in Mothur. Quality filtering was carried out using strict criteria of no ambiguous bases and no N bases; also, a minimum (412 bp) and maximum (426 bp) length of contig was applied. De novo analysis, using a 99% clustering similarity to form OTUs and assignment of staphylococcal species using a collated reference database of 37 staphylococcal species (see Additional file 2: Table S2) [5] at 97% similarity, was carried out in QIIME. The relative sequence abundance of each staphylococcus species composing the nasal staphylococcus microbial community was calculated for each sample. The presence of a staphylococcal species was determined using a >0.1% sequence abundance cut-off.

For calculation of alpha and beta diversity, de novo analysis using a 97% clustering similarity to form OTUs, and assignment of staphylococcal species using a collated reference database of 37 staphylococcal species at 97% similarity, was carried out in QIIME, and unassigned sequences were removed. Rarefaction was performed a depth at 1100 sequence reads.

## Results

### Study population

Eighteen patients were included in the study, six of which had received no antibiotics, six who received flucloxacillin (1 g) and gentamicin (160 mg) and six received teicoplanin (400 mg) +/− gentamicin (160 mg) (three patients received teicoplanin alone and three patients received teicoplanin in combination with gentamicin). No patients received nasal decolonisation treatment (mupirocin and chlorhedixine) prior to surgery, as all patients were MRSA negative by routine diagnostic screening. The patient demographic comprised of four males and 14 females, with age ranging from 52 to 91 years (median 81 years) (Table 1). A total of 94 swabs were taken, with a range of 3–7 days for each patient (mean = 5) (Table 1).

**Table 1:** Demographic summary of study participants

Antibiotic surgical prophylaxis received	Antibiotic dosage	Patient study ID	Age (years)	Sex	*S. aureus* nasal carriage detected by conventional culture on admission	Surgical speciality	Surgical procedure	Sample days
No antibiotics		25	79	Female	No	Orthopaedic	None	5
		99	52	Male	Yes	Cardiology	Angioplasty	4
		101	83	Female	No	Cardiology	Angioplasty	4
		105	63	Male	Yes	Cardiology	Angioplasty	5
		111	68	Female	Yes	Cardiology	Angioplasty	5
		113	79	Female	No	Cardiology	Angioplasty	3
Flucloxacillin (1 g) and gentamicin (160 mg)	Single dose IV on induction, and 2 further doses of flucloxacillin (1 g) post op at 6-h intervals	34	87	Female	No		Gamma Nail	7
		36	87	Female	No	Orthopaedic	Gamma Nail	7
		42	88	Female	Yes	Orthopaedic	Dynamic Hip Screw	7
		51	82	Female	No	Orthopaedic	Dynamic Hip Screw	5
		56	72	Female	No	Orthopaedic	Open Reduction of internal fracture of wrist and K wires	7
		61	88	Male	Yes	Orthopaedic	Total knee replacement	5
						Orthopaedic		
Teicoplanin (400 mg) +/− gentamicin (160 mg)	Single dose IV on induction	35^b^	80	Female	No	Orthopaedic	Hemiarthroplasty	7
		37^b^	91	Female	Yes	Orthopaedic	Hemiarthroplasty	7
		65^a^	76	Female	No	Orthopaedic	Total hip replacement	4
		86^b^	71	Male	Yes	Orthopaedic	Total hip replacement	4
		90^a^	81	Female	Yes	Orthopaedic	Hemiarthroplasty	5
		93^a^	89	Female	Yes	Orthopaedic	Hemiarthroplasty	4

^a^Teicoplanin alone

^b^Teicoplanin and gentamicin

### *Tuf* gene fragment amplicon sequencing

A total of 8,896,680 sequence reads were initially analysed from 94 samples, with the number of sequence reads ranging from 1084 to 600,830 per sample (median 71,991). Following quality filtering, 72 (76.6%) samples had >5000 sequence reads, and for these samples, 5000 representative sequence reads were subsampled; for the remaining samples, all the sequences were analysed (see Additional file 3: Table S3). After subsampling, 419,765 sequence reads remained to be analysed. Of the total sequence reads, 383,471 (91.4%) were assigned a staphylococcal species. Twenty-two staphylococcal species were identified in total, with 14 species identified from admission and subsequent samples and eight only on subsequent sampling during hospitalisation. All eight species identified from post-admission samples were present at <2% relative sequence abundance, with three species <0.1% relative abundance. *Staphylococcus epidermidis* (235,985, 56.2% of sequence reads) was the most dominant species. A total of 36,294 sequence reads were classified as unassigned. A BLAST search of unassigned sequences showed that the majority (85%) of unassigned sequences were from the staphylococcus genus, but species could not be assigned as identity was below the 97% threshold. A minority (15%) of unassigned sequences were identified as species of other Gram-positive bacteria including *Gemella haemolysans* and *Abiotrophia defectiva*.

### Investigation of the composition of the nasal staphylococcal microbiome in patients on admission

Fourteen staphylococcal species were identified by *tuf* gene fragment amplicon sequencing in the 18 nasal samples on admission (Table 2). *S. epidermidis* was the dominant species of the nasal staphylococcal microbiome and was carried by 100% of patients on admission (sequence abundance ranging from 0.74 to 94.60%). Thirteen patients were *S. aureus* nasal carriers on admission with sequence abundance ranging from 0.10 to 98.14%. *S* *taphylococcus capitis* and *Staphylococcus hominis* were present in over 75% of patients on admission, at median relative sequence abundance of 1.49 and 0.80%, respectively. The remaining staphylococci identified were present at low relative sequence abundance with medians <1.2%. The composition and diversity of the nasal staphylococcal microbiome varied from patient to patient on admission, with a range of 4 to 10 species present (average 6.5 species).

**Table 2:** Frequency of Staphylococcus species identified on admission and relative sequence abundance in study patients (*n* = 18)

	*tuf* fragment amplicon sequencing			
Staphylococcal species	Number of patients species was identified in	Percentage of patients species was identified in (%)	Relative sequence abundance	
			Median (%)	Range (%)
*S. aureus*	13	72.2	13.00	(0.21–98.14)
*S. auricularis*	4	22.2	0.54	(0.44–19.16)
*S. capitis*	16	88.9	1.49	(0.14–22.42)
*S. cohnii*	5	27.8	0.98	(0.10–2.60)
*S. epidermidis*	18	100.0	46.86	(0.74–94.60)
*S. haemolyticus*	7	38.9	0.76	(0.10–3.58)
*S. hominis*	14	77.8	0.80	(0.24–47.86)
*S. kloosii*	1	5.6	0.33	(0.33–0.33)
*S. lugdunensis*	8	44.4	0.24	(0.10–41.46)
*S. pasteuri*	7	38.9	0.57	(0.12–1.04)
*S. pettenkoferi*	7	38.9	1.18	(0.14–4.69)
*S. saccharolyticus*	4	22.2	1.05	(0.12–6.34)
*S. simulans*	3	16.7	0.22	(0.14–0.26)
*S. warneri*	11	61.1	0.53	(0.12–2.80)

On a sample by sample basis, the staphylococcal species identified from nasal samples by conventional culture and *tuf* gene fragment amplicon sequencing were compared. In 28.3% of instances, from the 18 samples, the same species was identified using both techniques, with 76.5% of these at a sequence abundance of >10% when analysed by *tuf* gene fragment amplicon sequencing. A significant proportion (70.0%) of species were only identified by *tuf* gene amplicon sequencing. Of the species with <10% sequence abundance, only 9.0% were also identified with conventional culture, with *Staphylococcus warneri* detected at the lowest sequence abundance (0.36%). There were only two instances of staphylococcal species being identified by conventional culture only (*Staphylococcus lugdunensis* in patient 86 and *Staphylococcus haemolyticus* patient 56). On admission, *tuf* gene fragment amplicon sequencing identified 13 patients with *S. aureus* carriage, compared to only 9 by conventional culture.

### Impact of antibiotic surgical prophylaxis on the nasal staphylococcal microbiome on alpha and beta species diversity

All samples were rarefied to a depth of 1100 sequence reads, as rarefaction curves of the Shannon index reached a plateau at a sequencing depth of less than 1100 reads (data not shown). Ninety nasal samples were analysed by alpha and beta diversity; four samples were excluded due to insufficient sequence reads depth. The alpha diversity of all 90 samples, measured by the Shannon index, ranged from 0.115 to 2.193 (median 1.094). On admission and throughout hospitalisation, the alpha diversity of nasal samples varied between individual patients irrespective of antibiotic administration. The extent of variation in alpha diversity between nasal samples taken from an individual patient varied, with a narrow diversity range in some patients (e.g. 1.517 to 1.917 Shannon index, patient 51) to a wide diversity range in other patients (0.726 to 2.193 Shannon index, patient 37). There was no significant difference in the alpha diversity between nasal samples taken on each sample day from patients in the three antibiotic regimens. Beta diversity was measured by the Bray Curtis dissimilarity. No substantial shift in the diversity of the nasal staphylococcal microbiome was observed after the administration of antibiotics when compared to admission sample (Table 3). Only one patient who received flucloxacillin and gentamicin (patient 51) had Bray Curtis dissimilarity greater than 0.5 for all four sample days compared to admission (0.77, 060, 0.65 and 0.78).

**Table 3:** The beta diversity of the nasal staphylococcal microbiome between admission and other sample days after administration of antibiotic surgical prophylaxis in individual study patients, measured by the Bray Curtis dissimilarity distance

Antibiotic surgical prophylaxis received	Patient study ID	Beta diversity (Bray Curtis dissimilarity) Sample day					
		2	3	4	5	6	7
No antibiotics	25	0.24	0.42	#	0.29		
	99	0.12	0.04	0.09			
	101	0.55	0.35	0.54			
	105	0.01	0.09	0.02	0.37		
	111	0.53	0.09	0.06	0.35		
	113	0.06	0.20				
Flucloxacillin (1 g) and gentamicin (160 mg)	34	0.23	0.21	0.20	0.20	0.21	#
	36	0.35	0.10	0.11	0.13	0.14	0.41
	42	0.25	0.19	0.66	0.33	0.70	0.54
	51	0.42	0.25	0.34			
	56	0.03	0.11	0.07	0.26	#	0.17
	61	0.77	0.60	0.65	0.78		
Teicoplanin (400 mg) +/− gentamicin (160 mg)	35^b^	0.21	0.33	0.33	0.24	#	0.17
	37^b^	0.30	0.06	0.22	0.32	0.40	0.20
	65^a^	0.08	0.08	0.06			
	86^b^	0.10	0.18	0.23			
	90^a^	0.06	0.08	0.05	0.36		
	93^a^	0.36	0.49	0.29			

^a^Teicoplanin alone

^b^Teicoplanin and gentamicin

^#^Beta diversity was not calculated for sample as excluded due to insufficient read depth (<1100)

### Impact of antibiotic surgical prophylaxis on the relative abundance of species present in the nasal staphylococcal microbiome

**Fig. 1:**
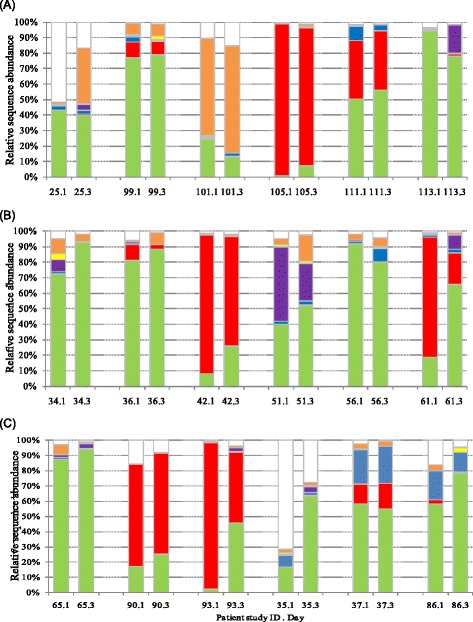
Relative sequence abundance of nasal staphylococcal microbiome on day 1 and day 3 of 18 patients who received antibiotic surgical prophylaxis regimen: **a** no antibiotics, **b** flucloxacillin and gentamicin, and **c** teicoplanin +/− gentamicin. *Colours* indicate *S. aureus* (*red*), *S. epidermidis* (*green*), *S. capitis* (*blue*), *S. hominis* (*purple*), *S. haemolyticus* (*yellow*), other staphylococci (*orange*), unassigned sequences (*white*). Number.number designation on *x*-axis represents the patient ID and sample day, with day 1 being pre-antibiotic treatment and day 3 being post-antibiotic treatment. Teicoplanin alone (patient study IDs 65, 90 and 93), teicoplanin and gentamicin (patient study IDs 35, 37 and 86)

To determine the impact of antibiotic surgical prophylaxis administration on the relative abundance of staphylococcal species present in the nose, samples from days 1 and 3 were compared. In patients who received no antibiotics, only minor changes in the relative sequence abundance of staphylococcus species occurred, with the majority of changes being an increase or decrease of <10% of a species (Fig. 1). More substantial changes in relative sequence abundance occurred in patients who received antibiotics. An increase in the sequence abundance of *S. epidermidis*, ranging from 6.7 to 47%, occurred in 10 out of 12 patients that received antibiotics. Of the nine *S. aureus* nasal carriers that received antibiotic surgical prophylaxis, eight had a reduced sequence abundance of *S. aureus* after the administration of antibiotics ranging from 0.2 to 57.4%. Although *S. aureus* relative sequence abundance was reduced, nasal carriage persisted. Six of the nine *S. aureus* carriers identified by amplicon sequencing were also identified by conventional culture. After the administration of antibiotics, *S. aureus* became undetectable by conventional culture in two patients but was still detectable by amplicon sequencing at low relative sequence abundance. A small reduction in the sequence abundance of *S. capitis*, *S. haemolyticus* and *S. hominis* was noted in the majority of patients who received antibiotics.

## Discussion

Application of a novel culture independent technique using the *tuf* gene has provided a more in-depth, detailed insight into the diversity of the nasal staphylococcal microbiome, and the impact of antibiotic surgical prophylaxis. Previous culture-dependent studies were restricted by their limit of detection, resulting in them being unable to reveal the true species diversity, especially at low abundance [12–14].

The novel use of the *tuf* gene in this culture independent study, as opposed to the more widely used 16S rRNA gene, has enabled the study of the nasal staphylococcal microbiome at species level [1–3, 12, 15–22]. Studies utilising the 16S rRNA gene have been unable to identify staphylococci at species level due to the high gene sequence similarity of 16S rRNA gene between staphylococcal species and thus described staphylococci either as Firmicutes staphylococcus genus, only identifying *S. aureus* to species level. Kaspar et al*.* [12] recently demonstrated this limitation, with only *S. aureus* being identified by both 16S rRNA and culture, with nine other staphylococcus species including *S. epidermidis* and *S. captis* only being identified to species level by culture.

The use and type of antibiotic agents used for surgical prophylaxis has changed over the last 20 years due to the increase in antibiotic resistance and changing practices. Most guidance on surgical prophylaxis now recommends the use of either a single dose of antibiotics or continuation for less than 24 h [23]. However, there are no studies that have used culture independent methods to investigate the impact of surgical prophylaxis on the human microbiome. A recent study looking at longer courses of antibiotics (5–10 days) on the salivary and faecal microbiome found very similar results to the current study in the salivary microbiome with it remaining relatively stable following antibiotics, whilst large changes were observed in the faecal microbiome [24]. The authors hypothesised that these differences may be due either to the pharmacokinetics of the antibiotics, or possibly that the populations in the salivary microbiome are more intrinsically resilient. Whilst the nasal microbiome is different to that of the salivary microbiome, it is still exposed to some of the same stresses such as changes in temperature, oxygen and physical disturbance and may mean that these populations are intrinsically more resilient.

The most significant change in the nasal staphylococcal microbiome after the administration of antibiotics was the increase in abundance of *S. epidermidis*, and the reduction but not eradication of *S. aureus* in nasal carriers. Within this study population of orthopaedic patients undergoing surgery involving implants, both these findings are clinically significant. In orthopaedic surgery, nasal carriage of *S. aureus* has been identified as the most important risk factor of developing SSI, with a relative risk of 8.9 [25]. Staphylococci are the leading cause of PJI with CNS causing 30–43% of infections, predominantly *S. epidermidis*, and *S. aureus* causing 12–23% [7]. Although PJIs occur in <1% of operations, the infections are difficult to treat involving additional surgery and prolonged antibiotic therapy, with a high morbidity. Other staphylococcal species present in the nose, for example *S. capitis* and *S. lugdunensis*, although less pathogenic than *S. aureus* and *S. epidermidis*, have been reported to cause PJI [26, 27], and infective endocarditis [28, 29].

Molecular methods for screening of S. aureus nasal carriage have been demonstrated to have a higher sensitivity than culture [30, 31]. Data from this study supports these findings, with only nine patients being identified as nasal carriers by culture as opposed to 13 by sequencing on admission. Due to the increased sensitivity of molecular methods, questions have been raised about false positives compared to culture methods; however, as this study is able to look at S. aureus relative abundance, it is able to provide evidence to demonstrate that it is not due to false positives.

*S. aureus* nasal carriage relative abundance was reduced to a low level by antibiotic surgical prophylaxis, which might be associated with a reduced risk of SSI, but it was not eradiated. Our finding may explain the higher *S. aureus* SSI rate, observed by Bode and colleagues, in patients who received surgical prophylaxis alone (8.4%), compared to patients who received prophylaxis in combination with *S. aureus* decolonisation with mupirocin and chlorhexidine prior to surgery (3.6%) [32].

This is the first culture independent study to use an alternative taxonomy gene to study the nasal staphylococcal microbiome in detail during surgical prophylaxis. The main limitation of the study is the small patient population analysed and its demographic bias of female patients; further investigation is required to determine the significance of these changes.

## Conclusions

*Tuf* gene fragment amplicon sequencing has enabled a detailed culture independent study of the impact of surgical prophylaxis on the nasal staphylococcal microbiome during surgical prophylaxis. As the new technique discriminates to staphylococcus species level, it could be used to study staphylococci rich microbial communities and expand our knowledge of staphylococcal diversity and the potential risk of infection in at risk patient groups.

## Additional files

Additional file 1: Table S1. Primer sequences used in the initial PCR step of *tuf* gene fragment amplicon sequencing. (DOC 35 kb)
Additional file 2: Table S2. Accession numbers of 37 species included in reference database. (DOC 27 kb)
Additional file 3: Table S3. Staphylococcal relative sequence abundance for all patient samples. (XLSX 23 kb)

## Abbreviations

CNS: Coagulase negative staphylococci
MALDI-TOF: Matrix-assisted laser desorption/ionisation time of flight
OTU: Operational taxonomic unit
PCR: Polymerase chain reaction
PJI: Prosthetic joint infections
QIIME: Quantitative insights into microbial ecology
SSI: Surgical site infections
