# Supplementary material for: Staphylococcal species heterogeneity in the nasal microbiome following antibiotic prophylaxis revealed by tuf gene deep sequencing
Source: Microbiome. 2016 Dec 2;4:63. doi: 10.1186/s40168-016-0210-1 (PMC5134057; doi:10.1186/s40168-016-0210-1)
Supplement: Additional file 1: Table S1. — Primer sequences used in the initial PCR step of tuf gene fragment amplicon sequencing. (DOC 35 kb) [file 40168_2016_210_MOESM1_ESM.doc]

Table S1. Primers sequences used in the initial PCR step of *tuf* gene fragment amplicon sequencing.

Forward/Reverse overhanging adapter

Heterogeneity spacer

Forward/Reverse Target specific region for *tuf* gene

Tuf_F_0bp TCGTCGGCAGCGTCAGATGTGTATAAGAGACAG GCCAGTTGAGGACGTATTCT

Tuf_F_1bp TCGTCGGCAGCGTCAGATGTGTATAAGAGACAG T GCCAGTTGAGGACGTATTCT

Tuf_F_2bp TCGTCGGCAGCGTCAGATGTGTATAAGAGACAG GT GCCAGTTGAGGACGTATTCT

Tuf_F_3bp TCGTCGGCAGCGTCAGATGTGTATAAGAGACAG CGA GCCAGTTGAGGACGTATTCT

Tuf_F_4bp TCGTCGGCAGCGTCAGATGTGTATAAGAGACAG ATGA GCCAGTTGAGGACGTATTCT

Tuf_F_5bp TCGTCGGCAGCGTCAGATGTGTATAAGAGACAG TGCGA GCCAGTTGAGGACGTATTCT

Tuf_F_6bp TCGTCGGCAGCGTCAGATGTGTATAAGAGACAG GAGTGG GCCAGTTGAGGACGTATTCT

Tuf_F_7bp TCGTCGGCAGCGTCAGATGTGTATAAGAGACAG CCTCTGG GCCAGTTGAGGACGTATTCT

Tuf_R_0bp GTCTCGTGGGCTCGGAGATGTGTATAAGAGACAG CCATTTCAGTACCTTCTGGTAA

Tuf_R_1bp GTCTCGTGGGCTCGGAGATGTGTATAAGAGACAG A CCATTTCAGTACCTTCTGGTAA

Tuf_R_2bp GTCTCGTGGGCTCGGAGATGTGTATAAGAGACAG TC CCATTTCAGTACCTTCTGGTAA

Tuf_R_3bp GTCTCGTGGGCTCGGAGATGTGTATAAGAGACAG CTA CCATTTCAGTACCTTCTGGTAA

Tuf_R_4bp GTCTCGTGGGCTCGGAGATGTGTATAAGAGACAG GATA CCATTTCAGTACCTTCTGGTAA

Tuf_R_5bp GTCTCGTGGGCTCGGAGATGTGTATAAGAGACAG ACTCA CCATTTCAGTACCTTCTGGTAA

Tuf_R_6bp GTCTCGTGGGCTCGGAGATGTGTATAAGAGACAG TTCTCT CCATTTCAGTACCTTCTGGTAA

Tuf_R_7bp GTCTCGTGGGCTCGGAGATGTGTATAAGAGACAG CACTTCT CCATTTCAGTACCTTCTGGTAA
