# Supplementary material for: Staphylococcal species heterogeneity in the nasal microbiome following antibiotic prophylaxis revealed by tuf gene deep sequencing
Source: Microbiome. 2016 Dec 2;4:63. doi: 10.1186/s40168-016-0210-1 (PMC5134057; doi:10.1186/s40168-016-0210-1)
Supplement: Additional file 2: Table S2. — Accession numbers of 37 species included in reference database. (DOC 27 kb) [file 40168_2016_210_MOESM2_ESM.doc]

Table S2. Accession numbers of 37 species included in reference database
